# The telomere-to-telomere genome of *Fragaria vesca* reveals the genomic evolution of *Fragaria* and the origin of cultivated octoploid strawberry

**DOI:** 10.1093/hr/uhad027

**Published:** 2023-02-20

**Authors:** Yuhan Zhou, Jinsong Xiong, Ziqiang Shu, Chao Dong, Tingting Gu, Pengchuan Sun, Shuang He, Mian Jiang, Zhiqiang Xia, Jiayu Xue, Wasi Ullah Khan, Fei Chen, Zong-Ming Cheng

**Affiliations:** College of Horticulture, Nanjing Agricultural University, Nanjing 210095, China; Hainan Yazhou Bay Seed Laboratory, Sanya 572024, China; College of Horticulture, Nanjing Agricultural University, Nanjing 210095, China; Wuhan Benagen Tech Solutions Company Limited, Wuhan, Hubei 430021, China; Hainan Yazhou Bay Seed Laboratory, Sanya 572024, China; College of Horticulture, Nanjing Agricultural University, Nanjing 210095, China; Key Laboratory of Bio-Resource and Eco-Environment of Ministry of Education & State Key Laboratory of Hydraulics & Mountain River Engineering, College of Life Sciences, Sichuan University, Chengdu 610065, China; College of Tropical Crops, Hainan University, Haikou 570228, China; Wuhan Benagen Tech Solutions Company Limited, Wuhan, Hubei 430021, China; College of Horticulture, Nanjing Agricultural University, Nanjing 210095, China; College of Tropical Crops, Hainan University, Haikou 570228, China; Sanya Nanfan Research Institute from Hainan University, Sanya 572025, China; College of Horticulture, Nanjing Agricultural University, Nanjing 210095, China; College of Tropical Crops, Hainan University, Haikou 570228, China; Hainan Yazhou Bay Seed Laboratory, Sanya 572024, China; College of Tropical Crops, Hainan University, Haikou 570228, China; Sanya Nanfan Research Institute from Hainan University, Sanya 572025, China; College of Horticulture, Nanjing Agricultural University, Nanjing 210095, China

## Abstract

*Fragaria vesca,* commonly known as wild or woodland strawberry, is the most widely distributed diploid *Fragaria* species and is native to Europe and Asia. Because of its small plant size, low heterozygosity, and relative ease of genetic transformation, *F. vesca* has been a model plant for fruit research since the publication of its Illumina-based genome in 2011. However, its genomic contribution to octoploid cultivated strawberry remains a long-standing question. Here, we *de novo* assembled and annotated a telomere-to-telomere, gap-free genome of *F. vesca* ‘Hawaii 4’, with all seven chromosomes assembled into single contigs, providing the highest completeness and assembly quality to date. The gap-free genome is 220 785 082 bp in length and encodes 36 173 protein-coding gene models, including 1153 newly annotated genes. All 14 telomeres and seven centromeres were annotated within the seven chromosomes. Among the three previously recognized wild diploid strawberry ancestors, *F. vesca*, *F. iinumae*, and *F. viridis*, phylogenomic analysis showed that *F. vesca* and *F. viridis* are the ancestors of the cultivated octoploid strawberry *F.* × *ananassa*, and *F. vesca* is its closest relative. Three subgenomes of *F.* × *ananassa* belong to the *F. vesca* group, and one is sister to *F. viridis*. We anticipate that this high-quality, telomere-to-telomere, gap-free *F. vesca* genome, combined with our phylogenomic inference of the origin of cultivated strawberry, will provide insight into the genomic evolution of *Fragaria* and facilitate strawberry genetics and molecular breeding.

## Introduction

A number of gapless, telomere-to-telomere (T2T) plant genomes have been assembled using ultra-long read sequencing technology, including those of arabidopsis (*Arabidopsis thaliana*) [1], rice (*Oryza sativa*) [2], water melon [3], kiwifruit [4], banana (*Musa acuminata*) [5], and bitter melon (*Momordica charantia*) [6]. The term ‘telomere-to-telomere’ has been used to describe high-quality, fully complete genome assemblies that include all centromeric and repetitive regions with high accuracy, continuity, and integrity [7]. Such assemblies, in particular their accurate reconstruction of repetitive regions, provide insight into the structure of centromeres and telomeres, enable annotation of more protein-coding genes, advance comparative genomics and evolutionary biology, and ultimately provide accurate genome sequences for use in genetic domestication and breeding [8].

*Fragaria vesca* is a diploid species (2*n* = 14) with small fruit and a wide distribution that is native to Europe and Asia. *F. vesca* has drawn the attention of the global strawberry research community because of its numerous useful traits, including self-compatibility, small genome size, low heterozygosity, abundant seed production, small plant size, diversity of forms, and amenability to *in vitro* manipulation [9]. As a result, *F. vesca* has been established as a diploid model system for strawberry research, and numerous genetic resources have been developed. A draft genome sequence of *F. vesca* cv. ‘Hawaii 4’ was released very early in 2011 (v1.0) [10], and a chromosome-level assembly based on Pacific Biosciences (PacBio) sequencing and optical mapping was reported in 2018 [11]. After manual curation and re-annotation, the improved v4.0.a2 annotation was published in 2019, providing a better resource for functional and comparative research on strawberries and their relatives [12]. Recently, different from the previously sequenced 4 ‘Hawaii 4’ accessions, the availability of the ‘Yellow Wonder’ reference genome has prompted another essential genetic resource building of *F. vesca* [13]. In addition, *F. vesca* has contributed subgenome material to the octoploid strawberry species *F*. × *ananassa*, and its genome therefore offers a useful and straightforward genetic and geographic contrast to the intricacies of octoploidy [14]. However, the current chromosome-level *F. vesca* genome still has a number of gaps and non-anchored contigs, indicating room for continued improvement.

To this end, we assembled a T2T high-quality genome of *F. vesca* using ultra-long Oxford Nanopore Technologies (ONT) and PacBio HiFi sequencing, bridging all remaining assembly gaps in the currently available reference genomes. The availability of a gap-free *F. vesca* genome has provided the first opportunity for analysis of its telomere and centromere regions, and we used multiple tools to identify unique genes and protein sequences in these previously ‘dark’ regions. In addition to a high-quality reference genome, we reconstructed a better karyotype of *Fragaria* species and investigated the karyotype evolutionary history of octoploid *F.* × *ananassa*.

## Results

### A telomere-to-telomere gap-free genome of *Fragaria vesca*

We generated ~32.67 Gb of ONT ultra-long sequencing reads, 27.31 Gb of PacBio HiFi reads, and 32.10 Gb of Illumina paired-end sequencing data for genome assembly. An additional 44.56 Gb of high-throughput chromatin capture (Hi-C) sequencing data were used to validate the genome assembly by comparing the assembly data with the scaffolding data. The N50 length of the HiFi reads was 12.8 kb and that of the ONT reads was 105 kb (Table 1, Supplementary Data Table S2).

**Table 1:** Genomic libraries used in assembly and annotation

**Library type**	**Tissue**	**Number of reads**	**Average** **read length (bp)**	**Number** **of bases (Gb)**
ONT	Leaf	312 929	10 439	32.67
PacBio HiFi	Leaf	2 139 796	1276	27.31
Hi-C	Leaf	296 885 274	150	44.56
Illumina	Leaf	213 991 280	150	32.10
Full-length RNA-seq (ONT)	Leaf, stem, runner	34 594 328	714.29	24.71

**Figure 1:**
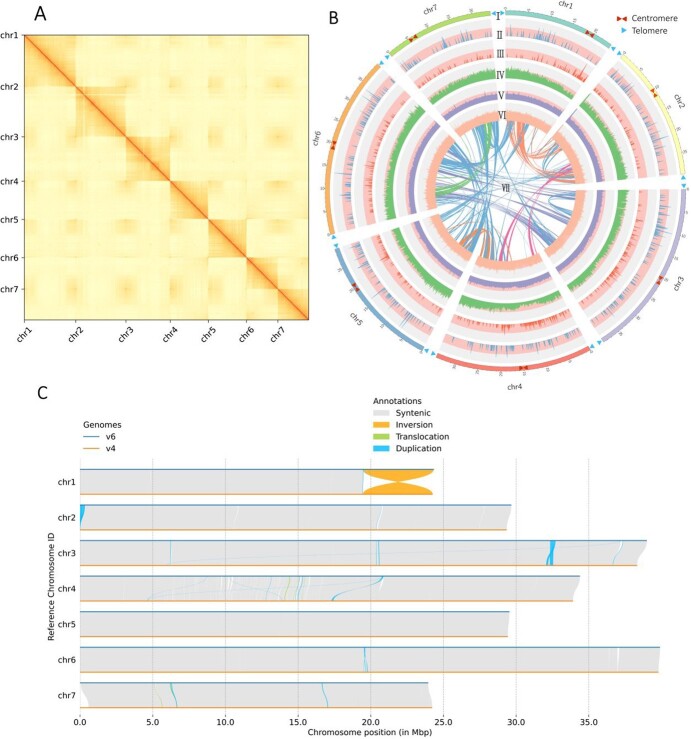
Complete genome assembly of *F. vesca*. (A) Hi-C interaction heat map showing that the *F. vesca* contigs were assembled into seven chromosomes. (B) Genomic features of *F. vesca.* I, seven chromosomes of *F. vesca*; II, density of *Copia* LTR–RTs; III, density of *Gypsy* LTR–RTs; IV, gene density; V, GC content density; VI, gene expression density; VII, syntenic blocks (all window sizes = 50 kb). (C) Structural variations between the v6.0 and v4.0 *F. vesca* genomes, using v6.0 as the reference. Non-syntenic regions indicate gaps in the v4.0 assembly.

We assessed *k*-mer-based quality (*k* = 21) using Illumina data (Supplementary Data Fig. S2). The ultra-long ONT and PacBio HiFi reads were assembled separately (see Materials and methods section). After the removal of non-nuclear sequences, we obtained 8 and 52 highly continuous contigs, respectively (Supplementary Data Table S2). Anchoring of contigs was performed (Fig. 1B), and the gap-free ONT genome was then used to fill gaps in the HiFi-assembled reference. Finally, a gap-free reference genome (v6.0) was created after all remaining gaps had been filled. The final genome was 220.8 Mb in length, longer than that of *F. vesca* v4.0, and had a contig N50 of 34.34 Mb (Table 2). The genome size of the v6.0 assembly was slightly lower than the estimate based on flow cytometry (~240 Mb), probably owing to bias in estimating a small genome size.

**Table 2:** Characteristics of the current genome assembly and previous assemblies

**Genomic feature**	**v6.0** This study	**v4.0** Edger *et al*., 2018 [11]	**v2.0** Tennessen *et al*., 2014 [15]	**V1.0** Shulaev *et al*., 2010 [10]
Genome size (Mb)	220.8	220.5	211.7	207.9
Contig N50 (Mb)	34.34	7.9		1.3 (scaffold N50)
Number of contigs	7	61	287	3200 scaffolds
Gaps	0	130	16 081	15 192
Number of telomeres	14	9	0	0
Number of centromeres	7	7	0	0
GC content (%)	38.5	38.35	35.69	34.5
Number of gene models	36 173	34 007 (v4.0.a2)	33 538 (v2.0.a2)	33 507 (v1.0 a2)
BUSCOs (%)	98.8	98.1 (v4.0.a2)	95.7 (v2.0.a2)	91.1 (v1.0 a2)

The high fidelity of the v6.0 assembly was supported by two high mapping rates of 99.5% (ONT) and 99.6% (Illumina) and two high coverages of 99.6% (ONT) and 95.4% (Illumina). BUSCO (Benchmarking Universal Single-Copy Orthologs) was used to evaluate genomic completeness, and 98.8% (*N* = 1614) of the conserved plant genes were identified and complete (Supplementary Data Table S3). By searching for the occurrence of the characteristic telomere motif (TTTAGGG) along the chromosomes, all 14 potential telomeric regions were revealed, containing a maximum of 216 and a minimum of 110 motif repeats. Likewise, the seven centromere regions were identified by searching for centromere proteins on each pseudochromosome (Fig. 1A).

We predicted 185 006 repetitive elements (78 313 685 bp), accounting for 35.63% of the v6.0 genome: 24.11% LTR-RTs, 9.29% uncharacterized TEs, and 2.23% DNA transposons (Supplementary Data Table S1). Using a combination of annotation methods, we predicted 36 173 genes in the *F. vesca* genome. The genomic sequences, coding sequences, exon sequences, and intron sequences had average lengths of ~3063, 1095, 312, and 407 bp, respectively (Supplementary Data Table S4). The set of 36 173 predicted protein-coding genes had a complete BUSCO recovery score of 98.8%, higher than any previous version of the strawberry genome. We also predicted 603 rRNAs, 484 tRNAs, and 405 snRNAs (Supplementary Data Table S6). A total of 32 101 (88.74%) protein-coding genes received annotations from at least one gene function database (Supplementary Data Table S5, Supplementary Data Fig. S3), such as the Gene Ontology (GO) database (58.35%). The number of predicted protein-coding genes was slightly lower in the v4.0a2 assembly (34 007), and the proportion of functionally annotated genes was also lower.

The v6.0 genome assembly had higher completeness and accuracy than the v4.0 assembly. In particular, all 130 gaps in the v4.0 assembly were successfully filled in this de novo assembly. Collinearity analysis showed 213 Mb of syntenic regions between the v6.0 and v4.0 genomes (Fig. 1C). A large inversion between the two genomes at the end of chr1 indicated that this region may have been arranged incorrectly in the older version. We also identified 594 structural rearrangements: 6 inversions, 20 translocations (91 021 bp), and 568 duplications (44 318 bp).

### Newly annotated genes in the complete genome of *Fragaria vesca*

**Figure 2:**
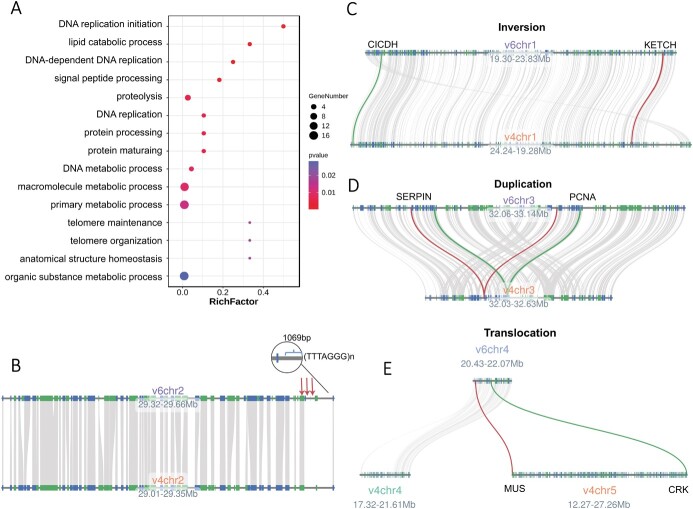
Newly annotated genes in the v6.0 version of the *F. vesca* genome compared with the v4.0a2 version. (A) GO annotations of the 1153 protein-coding genes present in the v6.0 assembly but absent from the v4.0 annotation. These genes are mainly involved in basic biological activities such as DNA replication, protein processing, and telomere organization. (B) The three newly annotated genes at the right end of chr2. The three red arrows represent the new genes, and the telomere repetitive sequence (1069 bp in total) is on the far right. (C) The inversion region on chr1 in v6.0. CICDH, cytosolic NADP-dependent isocitrate dehydrogenase. KETCH, karyopherin enabling the transport of cytoplasmic HYL1. (D) The duplicated region of chr3 in v6.0. SERPIN, serine protease inhibitor. PCNA, proliferating cell nuclear antigen. (E) The translocation region between chr4 and chr5 in v6.0. MUS, MUSTACHES. CRK, cysteine-rich receptor-like protein kinase.

When gene models from v6.0 were compared with those from v4.0.a2 [12], 26 165 clusters of genes were shared, accounting for 87.37% of v4.0.a1 and 73.45% of v4.0.a2. In total, 1153 genes were present in v6.0 but absent from v4.0.a2. We performed GO enrichment analysis to predict the functions of these newly annotated genes. The GO annotation results showed significant enrichment of genes related to fundamental biological processes such as telomere maintenance and organization and DNA replication in this gene set (Fig. 2A). Three newly annotated genes are located between positions 29.63 and 29.64 Mb of chr2, close to the telomere sequence (1069 bp) at the right end (Fig. 2B). There are more than 468 genes on the inversion cluster between 31.89 and 32.82 Mb on chr1, including *cytosolic NADP-dependent isocitrate dehydrogenase* (*CICDH*) and *karyopherin enabling the transport of the cytoplasmic HYL1* (*KETCH1*) (Fig. 2C). The 32.06–33.14 Mb duplicated region on chr3 contains numerous newly annotated genes, including those encoding a serine protease inhibitor (SERPIN) and proliferating cell nuclear antigen (PCNA) (Fig. 2D). In the translocated region between chromosomes 5 and 4, multiple genes originally on chromosome 5 of v4.0a2 are now annotated on chromosome 4 in v6.0, including genes encoding MUSTACHES (MUS) and a cysteine-rich receptor-like protein kinase (CRK) (Fig. 2E).

Higher plants have evolved a large number of cell-surface and intracellular immune receptors that sense various pathogen signals and promote resistance to pathogen invasion. One class of such intracellular receptors, the nucleotide-binding leucine-rich repeat (NLR) proteins, are frequently grouped within genomes, sometimes creating very large, rapidly evolving clusters of highly similar genes [16]. Here we used NLR-Annotator [17] software to identify 409 putative NLR loci, compared with 397 NLR loci in the v4.0a2 annotation (Supplementary Data Fig. S5). In addition, four *RCC1* (*Regulator of Chromosome Condensation 1*) genes are newly annotated in v6.0 (Supplementary Data Fig. S6).

### Telomere and centromere characteristics

Telomeres are fundamental conserved structures in plant genome sequences that typically consist of short, tandemly arranged minisatellites [18]. Here we identified the telomere regions in *F. vesca* and constructed a phylogenetic tree of *telomerase reverse transcriptase* (*TERT*) sequences from multiple plant species (Table 3, Supplementary Data Fig. S4). Telomerase is a ribonucleic acid–protein complex composed of telomerase RNA component (TERC) and TERT [19]. Its function is to synthesize telomeres at the ends of chromosomes, compensating for the gradual shortening of telomere length due to cell division and thus stabilizing the chromosomes (Supplementary Data Fig. S4B). A phylogenetic tree of the *TERT* gene sequence from 46 species, including 9 species of *Fragaria*, showed that its coding sequence is highly conserved (Supplementary Data Fig. S4C) and that it is maintained as a single-copy gene in most genomes. However, the natural allotetraploid *Nicotiana tabacum* [20] contains three sequence variants of the *TERT* gene, as does the octoploid cultivated strawberry *F. × ananassa*.

**Table 3:** Telomeres and centromeres in *F. vesca*

	**Telomeres**					**Centromeres**		
Chromosome	Left start	Left end	Right start	Right end	Right length (bp)	Start (bp)	End (bp)	Size (kb)
1	1	1880	24 344 918	24 346 798	920	19 510 000	19 520 000	10
2	1	918	29 669 392	29 670 488	1096	10 870 000	10 920 000	50
3	1	818	38 991 685	38 992 715	1030	20 440 000	20 460 000	20
4	1	1078	34 387 198	34 388 015	817	15 120 000	15 190 000	70
5	1	2075	29 535 993	29 536 839	846	19 650 000	19 680 000	30
6	1	877	39 893 091	39 893 988	897	19 680 000	19 690 000	10
7	1	975	23 953 275	23 954 239	964	5 160 000	5 290 000	130

In most eukaryotes, centromeric chromatin is composed of highly repetitive centromeric retrotransposons [21] (Supplementary Data Fig. S4A). We found that the centromeres of the seven *F. vesca* chromosomes were composed of a repeating 141-bp monomer (Supplementary Data 1).

### Evolution of the *Fragaria* chromosomes

The karyotype evolution of *Fragaria*—particularly that of cultivated strawberry and its three diploid wild relatives—has not previously been reported, and we therefore investigated the chromosome evolution of these species. The last common ancestor of the core eudicots had 7 ancestral chromosomes, and after the γ whole-genome triplication (WGT) event, 21 ancestral chromosomes (A1–A7, B1–B7, and C1–C7) became the basis for all core eudicots. Compared with the 21 chromosomes of the ancestral core eudicot karyotype, 19 genomic fission and 33 genomic fusion events gave rise to the current *F. iinumae* genome; 24 fission and 38 fusion events to the *F. viridis* genome; 17 fission and 31 fusion events to the *F. vesca* genome; and 141 fission and 155 fusion events to the *F.* × *ananassa* genome. We also estimated that 14 fission and 28 fusion events gave rise to the genome of *Rosa rugosa*. These results suggest that the *F. vesca* genome is the most conserved and stable among these *Fragaria* species, with the fewest genomic shuffling events after the γ WGT. Compared with the total fission and fusion events in the three diploid genomes, cultivated strawberry *F.* × *ananassa* had more fission and fusion events, implying that additional genomic reshuffling may have occurred after the ploidy fusion.

**Figure 3:**
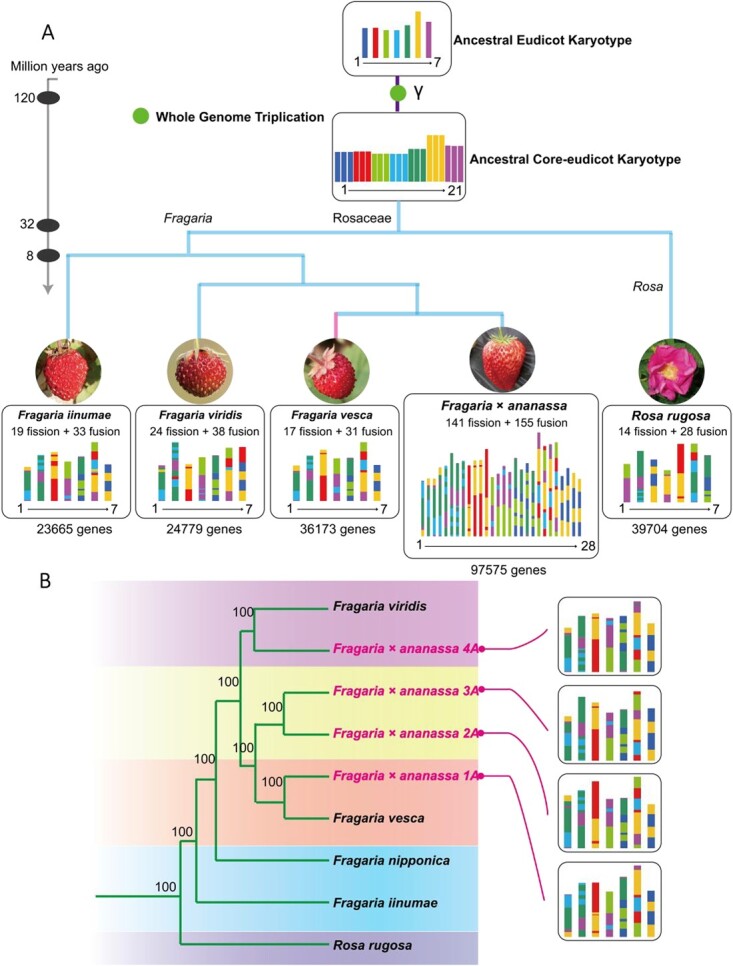
The contribution of *F. vesca* to cultivated octoploid strawberry. (A) Chromosome-level genomic evolution of three wild diploid strawberries and cultivated octoploid strawberry. Branch lengths represent divergence times. (B) Phylogenetic tree of *F. viridis*, *F. vesca*, *F. nipponica*, *F. iinumae*, *R. rugosa* and four subgenomes of *F. × ananassa* (1A–4A), indicating that *F. vesca* and *F. viridis* are the closest ancestors of *F. × ananassa*.

A phylogenetic tree based on 2751 low-copy nuclear genes firmly placed *F. vesca* as a sister lineage to *F.* × *ananassa* with 100% bootstrap support (Fig. 3A). We then divided the genome of cultivated *F.* × *ananassa* into four subgenomes, and phylogenomic inference unambiguously placed three subgenomes (1A, 2A, and 3A) as close relatives or sister to *F. vesca* and the fourth subgenome 4A as sister to *F. viridis*. These results imply that the two wild diploid strawberries *F. nipponica* and *F. iinumae* are not direct ancestors of cultivated strawberry. This subgenome analysis also supports an AA.AA.AA.BB model for the genome structure of *F.* × *ananassa*, in which the three AA subgenomes come from the *F. vesca* group and the BB subgenome from the *F. viridis* group.

## Discussion

To date, relatively few plant genomes and no Rosaceae genomes have been assembled with T2T levels of completeness and accuracy [8]. Although the *F. vesca* genome was first reported in 2011 [10] and later assembled at the chromosome level in 2018, its most recent assembly still includes 37 gaps with an average length of 621 bp. These gaps are located in or near highly repetitive regions, including centromeres, telomeres, 5S rDNA gene clusters, and nucleolar organizer regions with 45S rDNA [1]. Using a combination of ultra-long sequencing and Hi-C scaffolding technologies, we generated a gap-free genome assembly of *F. vesca*, including all telomeres and centromeres. Its completeness and accuracy will make this assembly useful for genomic research, molecular breeding, and precise genome editing in *Fragaria*.

The subgenomic contribution of wild diploid strawberry genomes to cultivated octoploid strawberry has long been a subject of debate. East Asia is the center of wild strawberry diversity, with most diploid strawberries and all tetraploid strawberries found in China. Modern cultivated strawberry (*F.* × *ananassa*) is a hetero-octoploid that arose in 18th century France from an accidental cross between the North American octoploid *Fragaria virginiana* and the South American octoploid *Fragaria chiloensis*. Edger *et al*. hypothesized that it was descended from four distinct diploid ancestors [woodland strawberry (*F. vesca*), rice marsh strawberry (*F. iinumae*), green strawberry (*F. viridis*), and Japanese strawberry (*F. nipponica*)], and the matter appeared to be settled [14]. Liston *et al.* re-analyzed the same set of data but came to a radically different conclusion [22]. They believed that there were only two extant ancestors of octoploid strawberry (*F. vesca* and *F. iinumae*), adding to the controversy over the diploid origin of cultivated strawberry. Previous phylogenomic studies have relied on older data that may not have fully represented the whole genomic evolutionary history of the genus. For example, only 24 single-copy nuclear genes were used for subgenomic analyses of *F.* × *ananassa* [23]. We are therefore confident in the greater accuracy of the current phylogenomic study, which made use of >2000 genes.

Even though our assembled genome is only 0.3 Mb bigger than the previous version, v6.0 is a complete genome that can be examined down to the chromosome level. We also offer a fresh approach to studying the evolution of species. It is certain that the evolutionary relationship of octoploid strawberries and even other polyploid strawberries will be more thoroughly verified with the decoding of complete genomes of various strawberry species. In summary, the gap-free *F. vesca* assembly reported here represents an important milestone in the assembly of diploid strawberry genome sequences. The complete genomic resource, together with our recently established strawberry genome database [24], will assist horticultural researchers in identifying genetic markers, investigating gene functions, and translating findings into genetic improvements in *Fragaria*.

## Materials and methods

### Plant materials and sequencing

At Nanjing Agricultural University in Jiangsu, China, the strawberry ‘Hawaii 4’ was planted (Supplementary Data Fig. S1). High-molecular-weight DNA was extracted using the CTAB technique for ultra-long ONT sequencing. We utilized the SQK-ULK001 kit to create a standard library after conducting quality checks with a NanoDrop One spectrophotometer (NanoDrop Technologies, Wilmington, DE, USA) and Qubit 3.0 Fluorometer (Life Technologies, Carlsbad, CA, USA). A PromethION sequencer was used for sequencing (Oxford Nanopore Technologies, Oxford, UK).

PacBio HiFi sequencing was performed using the QIAamp DNA Mini Kit/DNeasy Plant Mini Kit (Qiagen) for extracting genomic DNA from fresh leaves. Each SMRTbell library was constructed using the Pacific Biosciences SMRTbell Template Prep Kit 1.0. The constructed library was size­selected with the SageELF electrophoresis system to obtain molecules 11–15 or 14–17 kb in length, and this was followed by primer annealing and binding of SMRTbell templates to polymerases with the DNA Polymerase Binding Kit. On the PacBio Sequel II platform, the sequencing took 30 hours.

Using a Covaris ultrasonicator, 1 μg of genomic DNA extracted by the CTAB method was randomly fragmented for Illumina sequencing. We sequenced the final quality-checked libraries generated on the BGISEQ-500 platform using fragments with a typical size of 200–400 bp obtained from the Agencourt AMPure XP-Medium kit. DNA nanoballs (DNBs) with >300 copies were produced by rolling-cycle replication of single-stranded circular DNA molecules. High-density DNA nanochip technology was used to load the DNBs onto a patterned nanoarray, and combinatorial probe-anchor synthesis was used to produce paired-end 100-bp reads from the array. A total of 13 cycles of PCR were required to amplify the Hi-C libraries before sequencing on the HiSeq 2500 platform to produce 2150-bp reads.

The NEBNext Poly(A) mRNA Magnetic Isolation Module was used to enrich total RNA for poly(A) mRNA from root, leaf, and stalk tissues. The strand-switching method from Oxford Nanopore Technologies was used to create cDNA. In short, the Oxford Nanopore (SQK-PCS109) cDNA-PCR Sequencing Kit was used to create full-length cDNA libraries from the poly(A) mRNAs. Then, using specific barcoded adapters from the Oxford Nanopore PCR Barcoding kit (SQKPBK004), the cDNA was amplified by PCR for ~13 cycles. A 1D sequencing adaptor was ultimately ligated to the cDNA before putting it into a PromethION sequencer’s FLO-PRO002 R9.4.1 flow cell. The MinKNOW app was used to do the sequencing run.

### Genome assembly and assessment

An assembly of long (15 kb) and extremely accurate (>99%) HiFi reads was conducted using Hifiasm (version 0.16.1) [25] with default settings. The ONT data were put together using the NextDenovo program (https://github.com/Nextomics/NextDenovo) with the following settings: genome size = 220 Mb, read cutoff = 50 000, seed cutoff = 55 959, and seed depth = 45. The assemblies were polished using both Illumina and ONT reads with five iterative rounds and HiFi reads with three iterative rounds using the NextPolish (version 1.4.1) software [17] under the default parameters. The ONT genome assembly formed 9 contigs, and the PacBio assembly formed 202 contigs. Our search for organelle-associated sequences obtained from the National Center for Biotechnology Information (NCBI) was performed using BLAT (version 35), and then we removed the mitochondrial genome contig, which was the shortest contig (0.4% of the genome) in the ONT genome. Before anchoring the 202 contigs generated from the HiFi data, we removed 144 contigs through comparisons with the Nucleotide Sequence Database. Two sets of primary contig genomes were generated.

Hi-C data were used to anchor the contigs to chromosomes. After combining the two of seven contigs generated from ONT data with ALLHiC (version 0.9.8) [26], seven scaffolds representing seven pseudochromosomes were obtained. ALLHiC was then used to cluster, order, and orient the 58 remaining HiFi contigs. Then, 3D-DNA (version 180419) [27], Juicer (version 1.6) (https://github.com/aidenlab/juicer/wiki), and Juicebox (version 1.11.08) were used to generate the interaction file. The gap-free ONT genome was used to fill gaps in the genome generated by Hifiasm. Finally, a heat map of genomic interactions was plotted with HiCExplorer (version 3.6) [28].

BUSCO [29] was used to assess the completeness of the genome assembly, and Merqury (version 1.3) (https://github.com/marbl/merqury) was used to evaluate the consensus quality value and completeness. To estimate mapping rates, Illumina and Hi-C reads were mapped to the final assembly with bwa (version 0.7) (https://github.com/lh3/bwa), and ONT and HiFi reads were mapped with minimap2 (version 2.17) (https://github.com/lh3/minimap2).

### Identification of telomeres and centromeres

In most plants, telomere sequences consist of conserved, tandemly arranged minisatellites in the form (3′-TTTAGGG/5′-CCCTAAA)_n_ as described in the Telomere Database (http://telomerase.asu.edu/sequences_telomere.html). Telomeres were identified in the seven *F. vesca* pseudochromosomes as regions in which the characteristic motif was repeated more than five times [30]. Centromics software (https://github.com/ShuaiNIEgithub/Centromics) was used to identify centromeres. A high density of short tandem repeats and a low density of genes is typical of centromere regions, and we used these characteristics to identify continuous clusters with seven candidate centromeric tandem repeats that were present in the v6.0 genomic sequence but not the v1.0 sequence.

### Genome annotation

For the identification and classification of repetitive sequences, we used RepeatModeler (version open-1.0.11) [31] for *de novo* prediction and collected its output as a repeat library. The *de novo* and known repeat libraries were merged and used to predict repetitive sequences in the whole genome using RepeatMasker (version open-4.0.9, http://repeatmasker.org/) [32] with the parameters -nolow -no_is -norna -parallel 2. RepeatMasker (version 1.1.2) was then used to predict TE type with the parameters RepeatProteinMask -noLowSimple -pvalue 0.0001. Finally, we integrated all predicted repetitive sequences.

Protein-coding gene structures in the v6.0 genome were predicted using *ab initio*, homology-based, and RNA-seq-based approaches. Before *ab initio* prediction with Augustus (version 3.3) [33] and GlimmerHMM (version 3.0.4) [34], BUSCO (version 5.2.2) [29] was used to obtain the training sets. Exonerate (v2.2.0, https://github.com/nathanweeks/exonerate) was used for homology-based gene prediction after aligning the four previous protein sequence sets from *F. vesca* (v4.a1, v4.a2, v2.a1, and v2.a2) by tblastn (version 2.7.1). In parallel, an established annotation pipeline [HISAT2 (http://daehwankimlab.github.io/hisat2/) to StringTie (https://ccb.jhu.edu/software/stringtie) to TransDecoder (https://github.com/TransDecoder/TransDecoder)] was used to predict gene models using the transcriptome datasets. Maker (version 2.31.10) [35] was used to integrate all prediction results and generate a final set of gene models.

Protein-coding genes were predicted using three methods. KEGG (Kyoto Encyclopedia of Genes and Genomes) annotations [36] were obtained using DIAMOND (version 0.9.30) [37] and KOBAS (version 3.0) [38]; protein domain and GO term annotations were obtained using InterProScan [39]; and protein family annotations were obtained using hmmscan [40] (version 3.3.2) to search the Pfam database. The program cmscan in INFERNAL (version 1.1.2) [41] was used to identify rRNA, snRNA, and miRNA sequences using the Rfam database [42] with parameters -Z 747.66 —cut_ga —rfam —nohmmonly —cpu 15. tRNAscan (version 1.3.1) [43] was used to predict tRNA sequences.

### Genomic comparisons and karyotype inference

The complete v6.0 genome assembly was aligned pair-wise to the v4 genome using SyRI (version 1.63) to identify syntenic regions and structural variations (inversions, translocations, and duplications). Orthovenn2 (https://orthovenn2.bioinfotoolkits.net/) was used to generate a Venn diagram between v6.0 and v4.0 using an e-value of 1e−10. To annotate genes that were newly identified in v6.0, we performed GO analysis with InterProScan 5 (v5.47) to characterize gene functions according to biological process, cellular component, and molecular function terms (http://geneontology.org). We used the R package clusterProfiler to perform and visualize the GO enrichment analysis. We used jcvi (v1.1.19, MCscan for python) [44] to find new or different genes annotated in v6.0 compared with v4.0, including those in inversion, duplication, and translocation regions. Then, we used NLR-Annotator software (https://github.com/steuernb/NLR-Annotator) to find out the NLR loci. To identify NLR genes in v6.0, we searched the predicted proteome of v6.0 using hmmsearch in HMMER based on the seed NLR (PF00319) from the Pfam database.

Protein sets for the *F. iinumae* genome v1.0, *F. viridis* YNU genome v1.0, *F. nipponica* genome v1.0, and *F.* × *ananassa* FL15.89–25 genome v1.0 were obtained from the Genome Database for Rosaceae (GDR, https://www.rosaceae.org/), and that for *R. rugosa* was obtained from our established database, http://eplantftp.njau.edu.cn/ [45]. We constructed the ancestral angiosperm karyotype (AAK) through the -km subroutine of WGDI [46] and then used the proteins of the ancestral core eudicot karyotype (AEK) to infer the karyotypes of the five strawberry species and *R. rugosa*. Finally, according to the four subgenomes of *F*. × *ananassa*, we used the -a and -at parameters (https://wgdi.readthedocs.io/en/latest/index.html), and we used ASTRAL (v5.7.1) [47] to construct a subgenome coalescent tree. To calculate fission and fusion events, we first counted all the collinear color blocks, which could get all the splitting times, and then calculated the fusion and fission times according to the total number.

### Phylogenomic inference

OrthoFinder (v2.4.0) [48] was used to identify and align orthogroups in the five *Fragaria* species and *R. rugosa*. The alignment was used as input to IQ-TREE (v1.6.12) [49] to generate a phylogenetic tree, and the MCMCTree pipeline of PAML (v4.9) [50] was used to calculate the species divergence times. Known divergence times were downloaded from the TimeTree website (http://timetree.org/).

## Acknowledgements

Fei Chen acknowledges funding from the National Natural Science Foundation of China (32172614), a startup fund from Hainan University and a Hainan Province Science and Technology Special Fund (ZDYF2023XDNY050).

## Author contributions

Z.C. and F.C. designed and led this project. Y.Z., Z.S., Z.X., and M.J. assembled and annotated the genome. Y.Z., C.D., T.G., P.S., S.H., K.W., and J.X. analyzed the data. Y.Z. and F.C. wrote the draft manuscript. Z.C., J.X., and F.C. discussed and revised the draft. All authors have read and agreed to the published version of the manuscript.

## Data availability

All raw sequencing data generated in this project, including HiFi, Hi-C, Illumina, and ONT data, have been deposited at NCBI (https://www.ncbi.nlm.nih.gov/) under BioProject accession number PRJNA905123. The genome assembly and annotation data are available at our GDS database: http://eplant.njau.edu.cn/strawberry/.

## Conflict of interest

The authors declare that they have no conflicts of interest.

## Supplementary data

Supplementary data is available at *Horticulture Research* online.

## Supplementary Material

Web_Material_uhad027
