# Supplementary material for: The telomere-to-telomere genome of Fragaria vesca reveals the genomic evolution of Fragaria and the origin of cultivated octoploid strawberry
Source: Hortic Res. 2023 Feb 20;10(4):uhad027. doi: 10.1093/hr/uhad027 (PMC10116950; doi:10.1093/hr/uhad027)
Supplement: Web_Material_uhad027 [file web_material_uhad027.zip › Manuscript_ clean.docx]

**Supplementary information**

**Table S1. Repetitive elements in the Fragaria vesca genome assembly v6.0.**

**Table S2. Assembly summary and quality assessment.**

**Table S3. Completeness of the genome assembly based on BUSCO recovery scores.**

**Table S4. Protein-coding genes in the Fragaria vesca genome predicted by multiple methods.**

**Table S5. Numbers of predicted genes functionally annotated using various databases.**

**Table S6. Annotations of non-coding RNAs in the genome assembly.**

**Figure S1. Images of *Fragaria vesca*.**

**Figure S2. Estimation of genome size and heterozygosity using GenomeScope software.**

**Figure S3. UpSet diagram of all gene functional annotations, showing numbers of genes annotated by multiple databases.**

**Figure S4: Identification of centromeres and telomeres in the F. vesca genome.**

**Figure S5. Distribution and classification of NLR (nucleotide-binding leucine-rich repeat) genes in the *F. vesca* v6.0 genome assembly.**

**Figure S6. The phylogenetic tree of *RCC1* (*Regulator of Chromosome Condensation 1*) between v4.0a2 (FvH4) and v6 (FvesChr).**

**Supplementary data 1. Example of the 141-bp centromere repeat monomer.**
